# Development of Econophysics: A Biased Account and Perspective from Kolkata

**DOI:** 10.3390/e23020254

**Published:** 2021-02-23

**Authors:** Bikas K. Chakrabarti, Antika Sinha

**Affiliations:** 1 Saha Institute of Nuclear Physics, Kolkata 700064, India; antikasinha@gmail.com; 2 S. N. Bose National Center for Basic Sciences, Kolkata 700106, India; 3 Economic Research Unit, Indian Statistical Institute, Kolkata 700108, India; 4 Department of Computer Science, Asutosh College, Kolkata 700026, India

**Keywords:** traveling salesman problem, simulated annealing technique, kinetic exchange model, Gini index, Kolkata index, minority game, Kolkata Paise Restaurant problem

## Abstract

We present here a somewhat personalized account of the emergence of econophysics as an attractive research topic in physical, as well as social, sciences. After a rather detailed storytelling about our endeavors from Kolkata, we give a brief description of the main research achievements in a simple and non-technical language. We also briefly present, in technical language, a piece of our recent research result. We conclude our paper with a brief perspective.

## 1. Introduction

**Figure 1:**
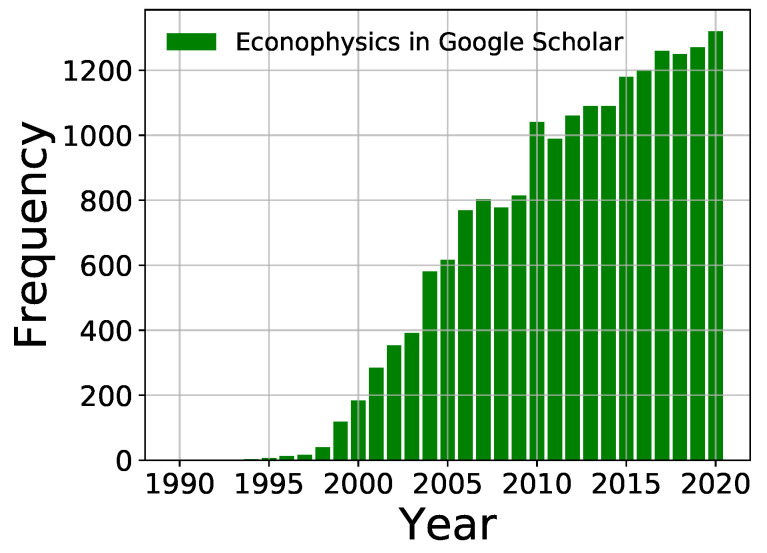
Histogram plot of yearwise numbers of entries containing the term econophysics against the corresponding year. The data are taken from Google Scholar (dated 31 December 2020). It may also be noted from Google Scholar that, while this 25-year old econophysics has today typical yearly citation frequency of order $1.3\times 10^{3}$, more than 100-year old subjects, like astrophysics (Meghnad Saha published his thermal ionization equation for solar chromosphere in 1920), biophysics (Karl Pearson coined the term in his 1892 book ‘Grammar of Science’), and geophysics (Issac Newton explained planetary motion, origin of tides, etc., in ‘Principia Mathematica’, 1687), today (31 December 2020) have typical yearly citation frequencies of order $32.5\times 10^{3}$, $26.8\times 10^{3}$, and $38.6\times 10^{3}$, respectively.

Countless attempts and research studies, mostly in physics, to model and comprehend the economic systems are about a century old. For the last three or four decades, major endeavor have been made and some successes have been achieved and published, notably under the general title ‘Econophysics’. The term was coined at a Kolkata conference held in 1995 by Eugene Stanley, who later in an interview said “ … So, he (Bikas) started to have meetings on econophysics and I think the first one was probably in 1995 (he decided to start it in 1993–1994). Probably the first meeting in my life on this field that I went to was this meeting. In that sense Kolkata is—you can say—the nest from which the chicken was born …” [1]. The entry on Econophysics by Berkeley Rosser in the New Palgrave Dictionary of Economics (2nd Edition [2]) starts with the sentence “According to Bikas Chakrabarti (…), the term ‘econophysics’ was neologized in 1995 at the second Statphys-Kolkata conference in Kolkata (formerly Calcutta), India, by the physicist H. Eugene Stanley …” See also Figure 1 (and Reference [3]). It may be mentioned here that in a more generalized sense, the term ‘Sociophysics’ was introduced more than a decade earlier by Serge Galam and coworkers [4] (also see Reference [5]).

As we will discuss in the next section, economics, like all the natural sciences (physics, chemistry, biology, geology, etc.), are, epistemologically speaking, knowledge or truth acquired through induction from observations (natural or controlled in the laboratories) using inductive logic and analyzed or comprehended using deductive logic (like mathematics). The divisions of natural sciences between the streams, like physics, chemistry, biology, and geology, are for convenience and are man-made. ‘Truth’ established in one branch or stream of natural science does not become ‘false’ or wrong in another; only the importance often vary. This helps in the growth of an younger branch of science through interdisciplinary fusion of established knowledge from another older established branch; astrophysics, geophysics, biophysics, and biochemistry had been earlier examples. Econophysics has been the latest one, and this special issue of Entropy attempts to capture the history, success and future prospect of econophysics research studies.

In the next five sections (essentially following the structure and section-titles, suggested by the Editors of this special issue), we discuss in non-technical language, allowing them to be accessible to the uninitiated younger students and researchers (except in Section 4, where we present some new result of our research). True to the spirit suggested by the Editors, the second section has been presented in the form of a ‘Dialogue’ using the format of questions and answers between us, the coauthors.

## 2. What Attracted You to Econophysics?

As mentioned earlier, this section is formatted in the form of a dialogue (question and answer) between the coauthors.

AS: What attracted you to Econophysics? Can you briefly recount?

BKC: Meghnad Saha, founder of Saha Institute of Nuclear Physics (named so after his death in 1956), had been a pioneering Astrophysicist (known for the Saha Ionization formula in astrophysics), had also thought deeply about the scientific foundation of many social issues (see, e.g., Reference [6]). In early seventies, our undergraduate-level text book on heat and thermodynamics had been ‘Treatise on Heat’ [7], written by Saha together with Biswambhar Nath Srivastava (first published in 1931). This turns out to be the earliest textbook where the students were encouraged, in the section on Maxwell-Boltzmann distribution in kinetic theory of ideal gas, to compare it with the anticipated ‘gamma’ distribution of income in the society (see the page from Reference [7] reproduced in Reference [6]). If taken seriously, it asks the students to model the income distribution in a society, which maximizes the entropy (assuming stochastic market transactions)!

AS: Before you go further, let me ask why should one think of applying statistical physics to society in the first place?

BKC: One Robinson Crusoe in an island cannot develop a running market or a functional society. A typical thermodynamic system, like a gas, contains Avogadro number (about $10^{23})$ of atoms (or molecules). Compared to this, the number (*N* ) of ‘social atoms’ or agents in any market or society is of course very small (say, about $10^{2}$ for a village market to about $10^{9}$ in a global market). Still such many-body dynamical systems are statistical in nature and statistical physical principles should be applicable. Remember, each constituents particle in a gas follows some well-defined equations of motion (say, Newton’s equation for classical gases or Schrödinger’s equation for quantum gases), yet for the collective behavior of gases (or liquids or solids) we need to average over the ‘appropriate’ statistics for their stochastic behavior in such ‘many-body’ systems and calculate the emerging collective or thermodynamic properties of the entire system. Motivation to go for the ‘appropriate’ statistics to estimate the collective behavior or response of the society comes, therefore, very naturally. In the case of human agents in a society, the corresponding equations governing individual behavior are much more difficult and still unknown and unpredictable, yet many collective social behavior are quite predictable; ask any traffic engineer or engineers designing stadium evacuation in panic situation.

AS: Which problem of economics did Saha and Srivastava try to analyze using Maxwell-Boltzmann distribution or statistical mechanics of ideal gas?

BKC: As can be seen from the example they had put to the readers, they indicated to the students the problem of income and wealth inequalities (they assumed Gamma-function-like income distribution in Reference [7]; reproduced in Reference [6]). They suggested to them that the ‘entropy maximization’ principle, along with conservation of money (or wealth), across the market (with millions of transactions between the agents, buyers, and sellers) must be at work in such ‘many-body’ social or market systems. This will result in the consequent and inevitable inequality (equal distributions being entropically unstable against stochastic fluctuations, leading to steady state unequal distributions).

AS: That is quite interesting. Can you elaborate a bit more and explain a bit of statistical physics specifically for the classical ideal gas?

BKC: Let me try. One can present the derivation of the energy distribution among the constituent (Newtonian) particles of a (classical) ideal gas in equilibrium at a temperature *T* as follows: If n(ϵ) represents the number density of particles (atoms or molecules of a gas) having energy ϵ, then one can write n(ϵ)dϵ=g(ϵ)f(ϵ,T)dϵ. Here, g(ϵ) denotes the ‘density of states’ giving g(ϵ)dϵ as the number of dynamical states possible for any of the free particles of the gas, having kinetic energy between ϵ and ϵ+dϵ (as counted by the different momentum vectors $\vec{p}$ corresponding to the same kinetic energy: ϵ= ${\left|p\right|}^{2}/2m$, where *m* denotes the mass of the particles).

Since the momentum $\vec{p}$ is a three-dimensional vector, $g\left(\epsilon \right)d\epsilon ∼{\left|p\right|}^{2}d\left|p\right|∼\left(\sqrt{\epsilon }\right)d\epsilon$. This is obtained purely from mechanics. For completely stochastic (ergodic) many-body dynamics or energy exchanges, maintaining the the energy conservation, the energy distribution function f(ϵ,T) should satisfy $f\left(\epsilon_{1}\right)f\left(\epsilon_{2}\right)=f\left(\epsilon_{1}+\epsilon_{2}\right)$ for any arbitrary choices of $\epsilon_{1}$ and $\epsilon_{2}$. This suggests f(ϵ)∼exp(−ϵ/Δ), where the factor Δ can be identified from the equation of state for the gas (positive sign in the exponential is neglected because of the observation that the number decreases with increasing energy). This gives n(ϵ)=g(ϵ)f(ϵ)∼ $\left(\sqrt{\epsilon }\right)\exp\left(-\epsilon /KT\right)$. Knowing this n(ϵ), one can estimate the average pressure *P* the gas exerts on the walls of the container having volume *V* at equilibrium temperature *T* and compare with the ideal gas equation of state PV=NKT (*K* denoting the Boltzmann constant). The gas pressure can be estimated from the average rate of momentum transfer by the atoms on the container wall, and one can compare with that obtained from the aforementioned equation of state and identify different quantities; in particular, one identifies Δ=KT.

AS: How does one then extend this to the markets?

BKC: Yes, let us consider the trading markets, where there is no production (growth) or decay. In addition, the total amount of money (considered equivalent to energy) and number of traders (or agents, considered as particles or ‘social atoms’) remain fixed or constant throughout the trades. Since in the market money remains conserved as no one can print money or destroy money (will end-up in jail in both cases) and the exchange of money in the market is completely random, one would again expect, for any buyer-seller transaction in the market, $f\left(m_{1}\right)f\left(m_{2}\right)$ $=f(m_{1}+m_{2})$, where f(m) denotes the equilibrium or steady state distribution of money *m* among the traders in the market. This then, in a similar way, suggests $f\left(m\right)∼\exp\left(-m/\Delta^{\prime }\right)$, where $\Delta^{\prime }$ is a constant. Since there cannot be any equivalent of the particle momentum vector for the agents in the market, the density of states g(m) here is a constant (any real-number value of *m* corresponds to one market state). Hence, the number n(m) of traders or agents having money *m* will be given by $n\left(m\right)=c\exp\left(-m/\Delta^{\prime }\right)$, where *c* is a constant. One must also have $\int_{0}^{M}n\left(m\right)dm$ =N, the total number of traders in the market, and $\int_{0}^{M}mn\left(m\right)dm=M$, the total amount money in circulation in the market (or country). This gives, the effective ‘temperature’ of the economy $\Delta^{\prime }=M/N$, the average available money per trader or agent in this closed-economy (as no growth, migration of laborers, etc., are considered). This gives exponentially decaying (or Gibbs-like) distribution of money in the market (unlike the Maxwell-Boltzmann or Gamma distribution of energy in the ideal gas), where most of the people become pauper (n(m) is maximum at m=0).

AS: Is this exponentially decaying income or wealth distribution realistic for any economy?

BKC: That discussion will take us to the recent studies by econophysicists and data comparisons. We will defer those to the next section (Section 3). Indeed, some success of the model (sketched above) in capturing the real data has been explored extensively by Victor Yakovenko and his group from Maryland University. We, in Kolkata, explored what could make the distribution more like a Gamma distribution, as Saha and Srivastava indicated in their book [6] to be an observed phenomenon. We also tried to capture the Pareto tail of such a distribution. Avoiding detailed discussion here, we only refer here to three popular papers [8,9,10] in this context. The model sketched above essentially follows [6,8]. In this model, the exchanged money or wealth in each trade (equivalent to any of the particle-particle collision in Ideal gas) is completely random, subject to conservation of money (or wealth). A trader, acquiring a lot in earlier trades may lose the entire amount of money or wealth in the next trade as the total money (wealth) will be conserved if the partner trader gets that. If one introduces a saving propensity of each trader, so that each trader saves a fraction of their individual money (wealth) before the trade and exchanges randomly the respective rest amount in the trade (keeping total money or wealth again conserved) the resulting steady state distributions capture the above mentioned desirable features. One can easily see that, unlike in the Kinetic-exchange model described above, the possibility for any trader (with non-vanishing saving propensity) to become an absolute popper vanishes, as that will require that trader to lose in every trade. Consequently, the exponential distribution becomes unstable with effect to any non-vanishing saving propensity and the stable distribution will become Gamma-like for uniform saving propensity of the traders [9] and initially Gamma-like but crossing over to Pareto-like power-law decay when traders have non-uniform saving propensities [10]. These results are non-perturbative results; any non-vanishing saving propensity will induce these features; the saving propensity magnitudes only determine the most-probable income (wealth) or the income (wealth) crossover point for Pareto tail of the distribution.

AS: Can we come back to your journey towards econophysics? Apart from Saha-Srivastava’s book, any influence from other books, especially from economics?

BKC: After Graduation and Post-Graduation from Calcutta University, I joined, in early 1975, the Saha Institute of Nuclear Physics as a Research Fellow in Condensed Matter Statistical Physics for my Ph.D. degree. By that time I had a huge personal collection of (mostly cheap editions, reprinted in India), general books, text books, other books and monographs in subjects outside physics; primarily in philosophy and economics. I had attempted closer studies of some them including: *The Problems of Philosophy*, Bertrand Russell (Cambridge Univ.), Oxford Univ. Press, Oxford (1959); *Mathematical Logic & the Foundations of Mathematics: An Introductory Survey*, Geoffrey Thomas Kneebone (Univ. London), D. van Nostrand Co. Ltd., London, UK, (1963); *The Problems of Philosophy*, Satischandra Chatterjee (Univ. Calcutta), Calcutta Univ. Press, Kolkata (1964); *The Philosophy of Wittgenstein*, George Pitcher (Princeton Univ.), Prentice-Hall Inc., New Delhi, India, (1964); *An Introduction to Philosophical Analysis*, John Hospers (Univ. Southern California), Prentice-Hall Inc., New Delhi (1971); Economics, Paul A. Samuelson (MIT), Tata-McGraw Hill, New Delhi (1971); and *Economic Theory & Operations Analysis*, William J. Baumol (Princeton Univ.), Prentice-Hall Inc., New Delhi (1978).

**Figure 2:**
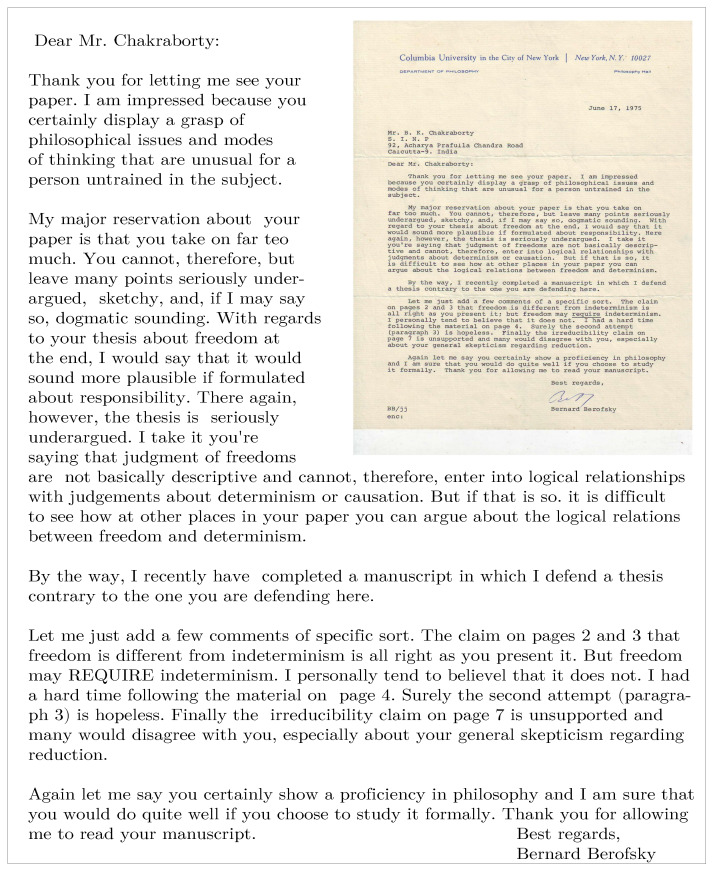
Reply (dated 17 June 1975) from Bernard Berofsky of the Philosophy Department of Columbia University to BKC on his criticisms of Bernard’s paper on ‘Indeterminism and Freedom’.

I tried to go through some of the isolated chapters or sections of these books, which I could understand, enjoyed, or liked most. Occasionally, I got excited and tried my own analysis, following them, on some interesting problems or discussions coming in my way. One such piece was a paper on ‘Indeterminism and Freedom’ by Bernard Berofsky of Columbia University, published in 1975, perhaps in Philosophical Quarterly. Among others, it also alluded to quantum physics in defending his thesis on freedom. I wrote a note detailing my criticisms and posted that to the author. The author, from the Department of Philosophy, Philosophy Hall, Columbia University in the City of New York, wrote to me the following letter on 17 June 1975 (see Figure 2):

AS: Obviously, you did not follow his suggestion, in fact, cordial invitation, to switch over to Philosophy. Why did you not?

BKC: Though I was seriously thinking of switching over to philosophy in a formal way, following Bernard’s suggestion, some quick apparent success in my physics research publications with the newly developed Renormalization Group theory in those days kind of blinded me and left me with two minds. Somehow, I failed to take a decision and continued with my physics research until I practically forgot about the other choice! In late 1978, I submitted my Ph.D. thesis in Condensed Matter Physics to the University of Calcutta and got the degree in 1979, and, by the end of that year, I left for post-doctoral research studies in the Theoretical Physics Department of the University of Oxford and the Institute of Theoretical Physics, University of Cologne.

I came back and joined the Saha Institute of Nuclear Physics as Lecturer in 1983, and I started my research in statistical physics with four Ph.D. students joining me simultaneously (including Subhrangshu Sekhar Manna, who later developed the ‘Manna Model’, belonging to the ‘Manna Universality Class’). Soon the statistical physics research in our group became so engaging and happening (with sixteen Ph.D. students, so far, getting their Ph.D. degrees and several of them becoming quite well known later for their pioneering research studies and still collaborating with me), I did not get much time until early nineties when I decided to try some research on ‘economics-inspired physics’. I went back to the problem Saha and Srivastava addressed in their textbook mentioned above and I co-organized a conference in January 1995, together with some established Indian statistical physicists and (reluctant!) economists as participants. In the Proceedings of the Conference, I published (together with an economist Sugata Marjit) my first paper [11] dealing with statistical physics of Income distribution and related problems.

By the end of the year, as a part of the StatPhys-Kolkata II (series of International Conferences organized by us in Kolkata every 3–4 years, latest event StatPhys-Kolkata X, held end of 2019), we had organized a special session on ‘Economics-Inspired Physics’ and Eugene Stanley in his talk coined the term ’Econophysics’ and had put that in the title of his paper [12] published in the Proceedings of the conference in Physica A, vol 224 (1996).

Though econophysics was quite risky as a topic of Ph.D. research in the late nineties (even today; still no faculty position in econophysics in our country, or for that matter, hardly exists elsewhere in the world), two brave students (Anirban Chakraborti and Arnab Chatterjee) expressed forcefully their desire to join the research on eventual topic of ‘econophysics’. I was also fortunate, my colleague Sitabhra Sinha also joined us in such investigations. In the last 25 years, since that conference, significant developments have taken place in the subject, and many of them will be covered this special issue of Entropy.

AS: We will come back to those developments later. I understand, most of the papers on econophysics research studies are published in physics journals and not in economics journals. What is the cultural level of appreciation by the intellectuals today?

**Figure 3:**
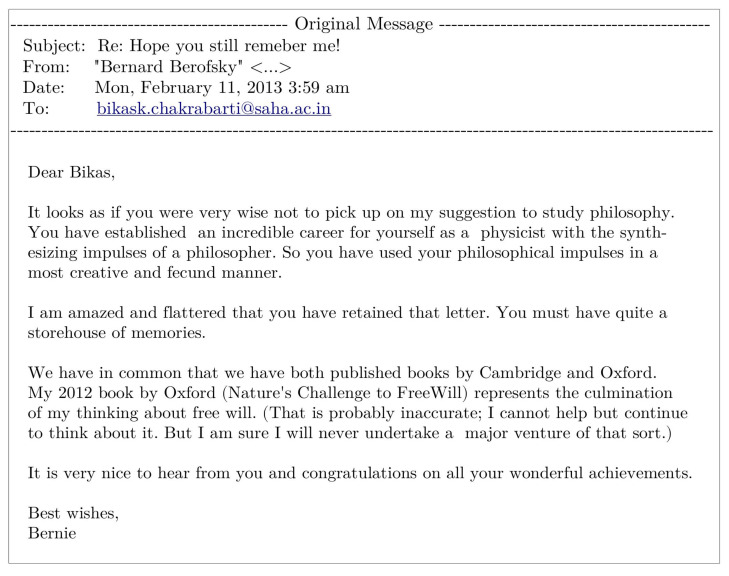
Mail from Bernard Berofsky of the Philosophy Department of Columbia University, in response to BKC’s surprise contact mail in 2013 (after almost thirty-seven years!), appreciating and identifying the development of econophysics as one due to the “physicist(s) with synthesizing impulses of a philosopher … (using) philosophical impulses in a most creative and fecund manner”.

BKC: This is indeed very difficult to answer. To tell very frankly, the response so far is not very supportive or encouraging! Although, it must be mentioned, the term econophysics has now entered in dictionaries of economics (see, e.g., Reference [2]) and Encyclopedias of social science and philosophy (see, e.g., Reference [13]). That brings me to an interesting, rather recent, correspondence with my old philosopher ‘guide’ Bernard Berofsky in January 2013, after thirty-seven years! This was quite accidental, when I came across in my internet search a new book published by him. I contacted him (giving a link to my homepage) saying, “sorry, I could not follow your advice so far and had been very shy to contact you. Now that I have become sixty, I acquired sufficient courage and …”. Bernard immediately responded (see Figure 3) praising the development of econophysics due to the philosophical impulses of physicists.

AS: Do you see really a philosophy behind econophysics?

BKC: Yes, indeed. I wrote about it earlier also (see, e.g., References [13,14]). I am not aware of all the documents on the mutual connection between philosophy and econophysics. I mentioned earlier about the entry on Econophysics in the Encyclopedia of Philosophy and Social Sciences [13]. I came to know of a rather recent entry on Social Ontology in The Stanford Encyclopedia of Philosophy [15] which, in the context of ‘social atomism’, writes “The idea is to model societies as large aggregates of people, much as liquids and gases are aggregates of molecules, …”. Then, after introducing the readers to two historical examples of Quetelet in 1848 [Adolphe Quetelet, 1848, Du système social et des lois qui le régissent, Paris: Guillaumin] and of Spencer in 1895 [Herbert Spencer, 1895, The Principles of Sociology, New York: Appleton] it says “Contemporary representatives include models in sociophysics and econophysics (see Chakrabarti et al., 2007) … [which] take a society or market to be an aggregate of these interacting individuals [Bikas K. Chakrabarti, Anirban Chakraborti and Arnab Chatterjee, 2007, Econophysics and Sociophysics: Trends and Perspectives, Hoboken, NJ: Wiley]”.

Let me now go back to the main to the main discussion and reiterate my basic argument in favor of considering economics as a natural science. Our knowledge about truth can, epistemologically speaking, be either deductive or inductive. Mathematics is an usual example of the deductive knowledge (though not all of it can be deduced from axiomatic logic); mathematical truths do not require any laboratory test or ‘observational’ support from the ‘nature’ to prove or validate them. Linguistically speaking, it is like the tautology “A bachelor does not have wife”. One does not need to check each and every bachelor to confirm truth of the statement—the first part of the sentence confirms the second part. The same is true about the statement “two plus two equals four”. Mathematical truths are analytical truths; left-hand side equals (in every intention and content) the right-hand side. Mathematics, therefore, is not directly a natural science [16,17,18], though it has been at the root of the logical structure of many natural sciences, particularly physics. Natural sciences, however, are basically inductive in nature. They are based on natural or (controlled) laboratory observations. The statement “The sun rises every twenty four hours on the east in the morning” is not a tautology nor an analytical truth. Though east may be defined as the direction, and morning may be defined by the time of sunrise, that it rises every twenty four hours is an inductive (or empirically observed) truth and, therefore, tentative (and not like mathematical truths, which are analytical and certain). Natural sciences start with observations and end in observations, using both inductive reasoning or logic; in-between, they often employ the tools of deductive logic, mathematics (as most condensed form of deductive logic).

AS: So, the tools of mathematics and logic are employed to find and establish relationships among these ‘natural’ observations to develop natural sciences. Where does then economics belong to?

BKC: That is the crucial question. Intermediate analysis using mathematics is just applied mathematics, and can not be considered as (pure) mathematics. Any branch of natural science does that. Economics has been and will be a part of natural science, where natural observations, not much of controlled or laboratory observations, need to be analyzed employing deductive logic and mathematics. Economics, therefore, should naturally belong to natural sciences!

AS: Agreed. But why econophysics?

BKC: You see, in natural sciences today, there are several branches or disciplines, like physics, chemistry, biology, geology, etc. The differences are not natural and certainly nature did not create them: they are human creations. The demarcations among these disciplines are not always clear. As we mentioned earlier, there are clear differences (in the nature of logic employed) between mathematics and natural sciences. But that does not extend to the branches of natural sciences. In a white light spectrum, our color perception continuously change from violet to red (without any sharp boundary) as the wavelength changes in this collection of electromagnetic waves. Similar are the cases of the different branches of natural science. They are not strictly differentiable; are historical in origin and continued by us for our own convenience (during upbringing; like perhaps religion; both are man-made). Of course, it is hard today to be an expert in the whole of even one branch of natural science. We, therefore, try to learn and acquire expertise in one sub-branch or a sub-sub-branch of natural science. An unique feature of the sub- or sub-sub-branches of natural science is that an established ‘truth’ or a ’fundamental law’ in one branch does not become ’false’ or ’wrong’ in another; only importance varies from discipline to discipline; quantum physics or gravity laws do not become invalid or wrong in chemistry or biology or mineralogy. Only gravity laws may be less important in chemistry or biology or mineralogy, and vice-versa. Models of geomagnetism in earth science cannot be built upon a law contradicting Maxwell’s laws of electromagnetism. Developments in younger branches in science, therefore, profitably utilized earlier established laws or ideas in older branches of natural science. Many of the early successful scientists (even some mathematicians) happen to have been identified as physicists, and, consequently, physics has become like an ‘elder brother’ among natural sciences, and it is now equipped with a huge armory of ideas, laws and models to comprehend the nature. Economics as a relatively newer entrant to natural science can, therefore, expect gainful advantages from such econophysical attempts!

AS: I remember you once told me that the concept of modeling dynamics of physical systems and of economics systems are fundamentally different. Can you elaborate the point in this context?

BKC: I do not remember which point we had been discussing. However, there is a typical one which may be discussed. Modeling dynamics of a physical system, like a particle, using, say, the Newton’s equation of motion, gives its dynamical state at a later time *t* by solving the equation of motion and utilizing the information regarding its dynamical state at an earlier time (say, at t=0; called initial conditions). Exact solutions may not be possible as in the thermodynamic or many-body systems, but based on the statistical characterization of the state of the system at an earlier time, the dynamical formulation helps solving the statistics of the system for any future time. The economic agents or organizations, even under nominally identical economic situation, may have (continually upgradeable) anticipation about the future and the model dynamics need to accommodate, along with their initial economic state, such anticipatory factors (which are continually adjusted or learned through the ongoing dynamics itself!) to solve for the future. Such self-consistent ‘learning’ dynamics of physical systems are not typical, though some recent many-body game theoretic models, with iterative learning for optimal use of scarce resources as in the binary-choice Minority Game (see, e.g., Reference [19]), or many-choice Kolkata Paise Restaurant Problem (see, e.g., Reference [20]) naturally incorporate such evolving learning features in the self-correcting dynamics themselves. We will discuss some details of the later problem here. In any case, these studies are new and still very limited in scope.

AS: To summarize, though many of you had started your econophysics research studies more than twenty five years back, since Gene Stanley coined the term econophysics in 1996 (in his publication [12] in the Proceedings of the second StatPhys-Kolkata Conference), and many more physicists joined after that, the subject is not established yet.

BKC: You are partly right. In fact, physicists have long been trying to formulate and comprehend various problems of economics. As mentioned before, since 1931, the statistical physics modeling of income and wealth distributions are being tried. However, these older physics attempts had been sporadic and isolated ones; physicists, successful in such attempts, like Jan Tinbergen (Economics Nobel Prize winner in 1969; had Ph.D. in statistical Physics under Paul Ehrenfest of Leiden University), had to migrate to economics department. Since 1996 (more correctly perhaps since 1991, when Rosario Mantegna published his paper [21] on Milan stock exchange data modeling), however, the situation has changed considerably. Physicists are now investigating economics problems along with their students and colleagues from the same department and are publishing their econophysical research papers in physics journals (in around 2000, Econophysics had been assigned the Physics and Astronomy Classification Scheme (PACS) number 89.65Gh by the American Institute of Physics).

I personally think, however, that an intensive and successful branch of econophysics research started with Scott Kirkpatrick and coworkers in 1983 when they proposed [22] the idea and technique of ‘Simulated Annealing’ (or ‘Classical Annealing’) to get practical solutions of the computationally hard multi-variable optimization problems, like the (managerial) economics problem of the Traveling Salesman Problem (TSP), using tuning (annealing) of Boltzmann-type fluctuations (simulating thermal ones) to escape from the local minima to reach eventually one of the (degenerate) global minima of the cost function (travel distance). This is a very successful story of the application of (statistical) physics to solve a problem which in nature and basic intent a (financial) economics problem involving multi-variable optimization. It may be noted in this connection that the technique has since been applied to all branches of science, as well as technology, and the original paper [22] has received major attention of scientists and engineers (so far having received more than 48,000 citations, according to Google Scholar). This idea still continues leading to a very intriguing and active domain of research in computationally hard problems of optimizations, using statistical physics and physics of spin glasses. This eventually led to the concept and technique of ‘Quantum Annealing’ (or of ‘Stochastic Quantum Computing’), where simulated quantum fluctuations (instead of simulated thermal fluctuations) are profitably used to tunnel through high but narrow local barriers [23], separating the global minima or solutions (see, e.g., Reference [24] for a brief review on solving TSP using quantum annealing). As I discussed earlier in my Econophysics-Kolkata Story [25], we started in 1986 (see Section 3.1) investigations on the statistical physics of the TSP. Soon my student Parangama Sen joined the effort [26]. (She eventually concentrated more on Sociophysics and developed, among others, the Biswas-Chatterjee-Sen model, see, e.g., Reference [27], for collective opinion formation together with our students Soumyajyoti Biswas and Arnab Chatterjee. In this connection, let me take the opportunity to acknowledge the contributions of my other students, Srutarshi Pradhan, Asim Ghosh and Sudip Mukherjee, Suchismita Banerjee, and, of course, you, Antika, and of my colleagues in the Kolkata-econophysics group, namely Anindya Sundar Chakrabarti, Manipushpak Mitra, and Satya Ranjan Chakravarty, allowing us to make some significant contributions to econophysics, which we are going to summarize in the next section.)

AS: So, you think that successful research studies in econophysics already started with the Simulated Annealing paper by Kirkpatrick et al. in 1983, although econophysics research studies on more popular economics problems started in 1990s and, more specifically, after Stanley coined the term in 1996?

BKC: Yes, you are right. We will discuss in little more details (in the next section; Section 3.1) the impact of statistical physics in developing the Simulated (Classical or Quantum) Annealing techniques for the financial computation problems involving multi-variable optimization of the Traveling Salesman type. The inspiring success of the classical annealing technique, initiated by the Simulated Annealing method, has led to several intriguing developments in statistical physics and to many applications in computer science. Further potential extension in the context of solving NP-hard problems using quantum annealing has become one of the core research topic today in quantum many-body (statistical) physics and in quantum computation. Indeed I consider this outstanding development of simulated (classical or quantum) annealing techniques (starting with Kirkpatrick et al. [22]; also see Reference [23]) for the Traveling Salesman type multi-variable optimization problems to be a landmark achievement in the true spirit of econophysics. Of course, the present phase of econophysics research activities stemmed from several influential papers, analyzing financial market fluctuations, by Rosario Mantegna and Eugene Stanley and in particular following the publication of Kolkata Conference Proceedings paper [12] by Stanley et al. in 1996.

## 3. Major Achievements and Publications of the ‘Kolkata School’

Physicist Victor Yakovenko and economist J. Barkley Rosser in their pioneering interdisciplinary collaborative review [28] in the Reviews of Modern Physics (2009) on econophysics of income and wealth distributions, discussed about some of the ‘influential’ and ‘elegant’ papers from the ‘Kolkata School’. We will briefly summarize in this section some of our major research studies in econophysics (including those on wealth distributions).

### 3.1. Traveling Salesman Problem and Simulated (Classical & Quantum) Annealing

As already discussed, the Traveling Salesman Problem or TSP is, in its intent and structure, a computationally involved financial management problem (see, e.g., References [29,30]). The problem can be easily defined as a geometric one. Suppose in an unit square area there are *N* random dots, representing the cities. The salesman has to make a visit to all the cities and come back with minimum travel cost. The travel cost to visit all these cities will depend on the total travel distance of the tour. Each component of the travel distance between any two cities can be taken as the Euclidean distance (or as appropriate for the spatial metric, say Cartesian) between them. One can easily check that there are N!/2 (growing faster than exponential in *N*) distinct tours or trips to visit all the *N* cities. Obviously, all of these trips do not have identical value (‘cost’) for the total travel length (*D*), and the problem is to find the trips(s) which will correspond to the minimum travel distance *D*. Searching over all the possible trips soon becomes impossible as *N* becomes large, and there is no perturbative way to improve on any randomly chosen travel path to reach the global solution. At any point or city on a tour, there are *N* order choices for the next move or visit and the optimization problem of the total travel distance is truly a multi-variable one. It may be noted that the problem becomes trivial in one dimension (homes or offices placed randomly on a straight road), where the salesman can start from one end of the road and finish at the other end). Generally, for two dimension onwards, search time for such a minimum ‘cost’ (from among exp(N) number of trips or configurations), cannot be bounded by any (deterministic) polynomial in *N* (NP-hard problem).

From now onwards, let us concentrate our discussion on TSP in two dimension. The scale of the total travel distance, however, can be easily guessed using a ‘mean field’ picture. If *N* randomly placed points (cities) fill an unit (normalized) area, then the ‘average’ or ‘mean’ area per city is 1/N, giving nearest neighbor distance to be of order $1/\sqrt{N}$ and total travel distance $D=\Omega \sqrt{N}$. Numerical estimates suggests Ω≃0.71 [31].

The problem is truly global in nature. Choice of the next city to visit depends on the position of even the farthest city in the country. However, one can approximately solve the problem (see References [32,33]) by reducing it to an effective one-dimensional problem where the country (unit square) is divided into hypothetical parallel strips of width *w* and the salesman visits the cities within each strip in a ‘directed’ way and the total travel distance *D* is optimized with respects to single variable *w* (optimal value then grows as $\sqrt{N}$) and gives (see, e.g., References [32,33]) Ω≃0.92. Another way is to put the cities randomly with concentration ρ on the lattice sites of, say, an unit square lattice. The lattice constraint can help then the calculation of the optimal travel distance. The optimal (normalized) travel path length then scales as $D=\Omega \sqrt{\rho }$. At ρ=1, the lattice constraints would immediately imply that the global search problem reduce to a local one and all the space filling Hamiltonian walks would correspond to optimized tour with Ω=1. In the approximate single variable solution (minimization of *D* with respect to *w*) indicated above, the strip width *w* grows as $1/\sqrt{\rho }$ as ρ decreases. For ρ→0, however, the lattice constraints disappear, and the problem reduces to TSP on continuum as defined earlier (NP-hard, w→∞, with Ω≃0.71 [31]). Where does the problem become NP-hard? This study was initiated by us (see References [26,34,35,36,37]) and they indicated (also Reference [32]) that the TSP on dilute lattices becomes NP-hard only at ρ→0 (though this is not settled yet and some arguments support that it crosses over to NP-hardness at $\rho =1_{-}$ or as soon as ρ becomes less than unity).

As already mentioned earlier (in Section 2), a major computational breakthrough of TSP and other such multi-variable optimization problems came from the 1983 seminal paper on Simulated Annealing’ [22] by Kirkpatrick et al., who proposed a novel stochastic technique, inspired by the metallurgical annealing process and statistical physics of frustrated systems.

Imagine a bowl on the table, and you need to ‘locate’ its bottom point. Of course, one can calculate the local depths (from a reference height) everywhere along the inner volume of the bowl and search for the point where the local depth is maximum. However, as every one would easily guess, a much simpler and practical method would be to allow rolling of a marble ball along the inner surface of the bowl and wait for locating its resting position. Here, the physics of the forces of gravity and friction allows us to ‘calculate’ the location of the bottom point in an analog way! Algorithm-wise, it is simple. For any possible move, if the changed ‘cost’ function has lower value, one should accept the move and reject it otherwise. Success for the search of the minimum is guaranteed. In principle, a similar trick would work for cases where the bowl becomes larger and its internal surface gets modulated, as long as the surface contour or ‘landscape’ has valleys all tilted towards the same bottom point location. Computationally hard problems arise when these valleys are separated by ‘barriers’, which are (macroscopically) high. The simulated annealing suggests a way out to overcome (at least for finite height barriers) by allowing moves costing higher to have (Gibbs-like) lower probability of acceptance.

To search for the optimized cost (travel distance in TSP or energy of the ground state(s) in spin glasses) at eventually vanishing level of noise (or ‘simulated temperature’), one starts from a high noise (temperature) ‘melt’ phase, and tune slowly the noise level. In this ‘simulated’ process, the (classical) noise at any intermediate level of annealing allows for the acceptance of the changed ‘costs’ ΔD in distance or energy *D*: 100% acceptance of the move if ΔD<0 and acceptance of the move with a Gibbs-like probability ∼exp(−ΔD/T) for moves with increased in cost (ΔD>0)). As the noise level (*T*) is slowly reduced during the annealing process, the gradually decreasing probability of accepting higher cost values, allows the system to come out of the local minima valleys and settle eventually in (one of) the ‘ground state’ (with minimum *D*) of the system. For slow enough decrease of noise T(t) with time *t*, one can estimate the quasi-equilibrium (thermal) average of the cost function <D> at ant time *t* and derive the effective ‘specific heat’ value δ<D>/δT as a function of *t*. One needs to be very slow (|dT/dt| very small) when the effective specific heat increases with decreasing *T*, indicating the ‘glass’ transition point and anneal at faster rates on both sides of the transition point.

As has been indicated in the earlier section, it has been a remarkably successful trick for ‘practical’ computational solutions of a large class of multi-variable optimization problems, as in most multi-city travel cost optimizations and similar multi-variable optimizations (see, e.g., References [29,30,38,39]).

Though some ‘reasonable’ optimization can be achieved very quickly using appropriate annealing schedules, the search time for reaching the lowest cost state or configuration for NP-hard problems, however, grows still as exp(N). The bottleneck could be identified soon. Extensive study of the dynamics of frustrated random systems, like the *N* spin (two state Ising) glasses, particularly of the Sherrington-Kirkpatrick variety (see, e.g., Reference [23] also for a TSP version of the quantum annealing), showed that its (free) energy landscape (in the ‘glass’ phase), is extremely rugged, and the barriers, separating the local valleys, often become *N* order implying the search for the degenerate ground states from $2^{N}$ (or N!/2) states is NP-hard (for the *N*-city TSP). In the macroscopic size limit (*N* approaching infinity), therefore, such systems effectively become non-ergodic or localized, and the classical (thermal) fluctuations, like that in the simulated annealing, fail to help the system to come out of such high barriers (at random locations or configurations, not dictated by any symmetry) as the escape probability is of order exp(−N/T) only. Naturally, the annealing time (inversely proportional to the escape probability), to get the ground state of the *N*-spin Sherrington-Kirkpatrick model, cannot be bounded by any polynomial in *N*.

**Figure 4:**
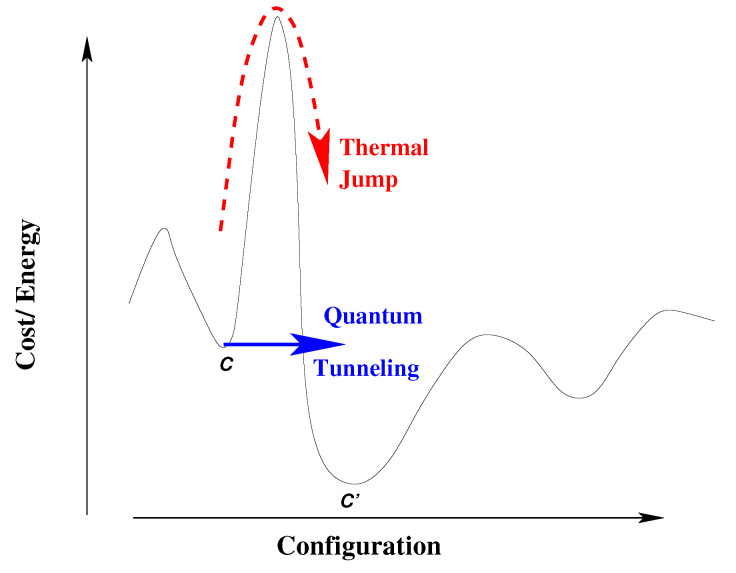
While optimizing the cost function of a computationally hard problem (like the minimum travel distance for the Traveling Salesman Problem (TSP)), one has to get out of a shallower local minimum, like the configuration C (travel route), to reach a deeper minimum C’. This requires jumps or tunneling, like fluctuations, in the dynamics. Classically, one has to jump over the energy or the cost barriers separating them, while quantum mechanically one can tunnel through the same. If the barrier is high enough, thermal jump becomes very difficult. However, if the barrier is narrow enough, quantum tunneling often becomes quite easy. Indeed, assuming the tall barrier to be of height *N* and width $\tilde{w}$, one can estimate (see, e.g., Reference [42]) the tunneling probability through the barrier to be of order $\exp[-\left(\tilde{w}\sqrt{N}\right)/\Gamma ]$, where Γ denotes the strength of quantum fluctuations (instead of the the classical escape probability of order exp[−N/T], *T* denoting the thermal or classical fluctuation strength).

The idea proposed by Ray et al. [40] was that quantum fluctuations in the Sherrington-Kirkpatrick model can perhaps lead to some escape routes to ergodicity or quantum fluctuation induced delocalization (at least in the low temperature region of the spin glass phase) by allowing tunneling through such macroscopically tall but thin (free energy or cost functions) barriers which are difficult to scale using classical fluctuations. This is based on the observation that escape probability due to quantum tunneling, from a valley with single barrier of height *N* and width $\tilde{w}$, scales as $\exp(-\sqrt{N}\tilde{w}/\Gamma )$, where Γ represents the quantum (or tunneling) fluctuation strength (see Figure 4). This extra handle through the barrier width $\tilde{w}$ (absent in the classical escape probability of order exp(−N/T)) can help in a major way in its vanishing limit. Indeed, for a single narrow ($\tilde{w}\to 0$) barrier of height *N*, when Γ is slowly tuned to zero, the annealing time to reach the ground state or optimized cost, will become *N* independent (even in the N→∞ limit; δ-function barriers are transparent to quantum fluctuations, while classical or thermal annealing to escape from such a barrier is impossible)! It has led to some important clues. Of course, complications (localization) may still arise for many such barriers at random ‘locations’. In any case, with this observation and some more developments, the quantum annealing technique was finally launched through the subsequent publications of a series of landmark papers (both theoretical and experimental; see Reference [23]) and through a remarkable practical realization of the quantum annealers by the D-wave Group [41].

Let us now conclude this subsection. Simulated Annealing technique, invented by Kirkpatrick et al. in 1983 [22], has since been applied extensively also to solve problems of collective decision making in economics and social sciences (see, e.g., Reference [43] for a recent review). As mentioned earlier [25], our group started investigations on statistical physics of TSP in 1986. The intriguing physics of Simulated Annealing inspired us to explore the possible further advantages of quantum tunneling (to allow escape through macroscopically tall but thin barriers in some NP-hard cases), where classical annealing (using thermal fluctuations) fails. This led finally to the quantum extension or to the invention of the quantum annealing technique, where our initial contributions (References [23,40]) are considered to be important and pioneering. See, e.g., Reference [24] for a brief review and Reference [44] for some recent discussions on the advantages of applying quantum annealing method to solve TSP. Quantum annealing is a very active research field today in quantum statistical physics and computation (see, e.g., References [45,46] for recent reviews).

### 3.2. Social Inequality Measure and Kolkata Index

Social inequality, particularly income or wealth inequality in, are ubiquitous. There are several indices or coefficients, used to measure them, the oldest and most popular one being the Gini index [47].

**Figure 5:**
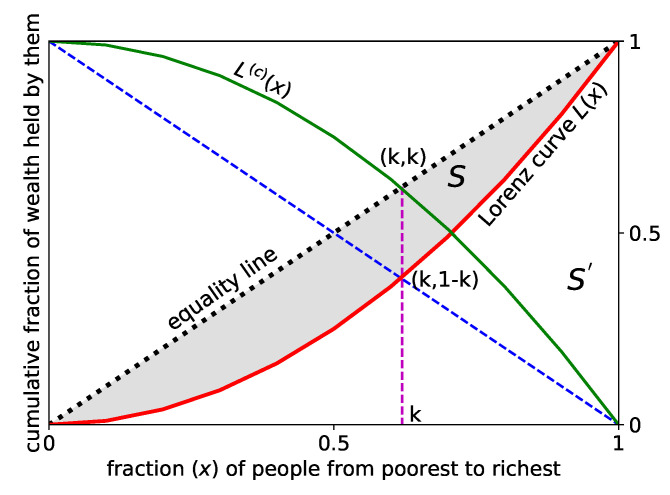
Lorenz curve (in red) or function L(x) here represents the fraction of accumulated wealth against the fraction *x* of people possessing that, when arranged from the poorest to the richest. The diagonal from the origin represents the equality line. The Gini index (g) can be measured by the area (S) between the Lorenz curve and the equality line (shaded region), normalized by the total area $\left(S+S^{\prime }=1/2\right)$ under the equality line: g=2S. The complementary Lorenz function $L^{\left(c\right)}\left(x\right)\equiv 1-L\left(x\right)$ is shown by the green line. The Kolkata index (k) can be measured by the ordinate value of the intersecting point of the Lorenz curve and the diagonal perpendicular to the equality line. By construction, $L^{\left(c\right)}\left(k\right)$ = 1−L(k)=k, saying that *k* fraction of wealth is being possessed by (1−k) fraction of richest population.

It is based on the Lorenz curve or function [48] L(x), giving the cumulative fraction of (total accumulated) income or wealth possessed by the fraction (*x*) of the population, when counted from the poorest to the richest (see Figure 5). If the income (wealth) of every one would be identical, then L(x) would be a straight line (diagonal) passing through the origin. This diagonal is called the equality line. The Gini coefficient (*g*) is given by the area between the Lorenz curve and the equality line (normalized by the area under the equality line: g=0 corresponds to equality and g=1 corresponds to extreme inequality.

We proposed [49] the Kolkata index or *k*-index given by the ordinate value of the intersecting point of the Lorenz curve and the diagonal perpendicular to the equality line (also see References [50,51,52,53,54]). By construction, 1−L(k)=k, saying that *k* fraction of wealth is being possessed by (1−k) fraction of the richest population. As such, it gives a quantitative generalization of the approximately established (phenomenological) 80–20 law of Pareto [55], saying that, in any economy, typically about 80% wealth is possessed by only 20% of the richest population. Defining the complementary Lorenz function $L^{\left(c\right)}\left(x\right)\equiv \left[1-L\left(x\right)\right]$, one gets *k* as its (nontrivial) fixed point (while Lorenz function L(x) itself has trivial fixed points at x= 0 and 1). *k*-index can also be viewed as the normalized *h*-index [56] for social inequality; *h*-index is given by the fixed point value of the nonlinear citation function against the number of publications of individual researchers. We have studied the mathematical structure of *k*-index in Reference [53] (see Reference [54] for a recent review) and its suitability, compared with the Gini and other inequality indices or measures, in the context of different social statistics, in References [49,50,51,52]. In addition, see Reference [57] for redefining a generalized Gini index and Reference [58] for a recent application in characterizing the statistics of the spreading dynamics of COVID-19 pandemic in congested towns and slums of the developing world.

In summary, inspired by the observations of richer structure (self-similarity) of the (nonlinear) Renormalization Group equations near the fixed point (see, e.g., Reference [59]), or of the nonlinear chaos-driving maps near the fixed point (see e.g., Reference [60]) and noting that inequality functions, such as the Lorenz function L(x) or the Complementary Lorenz function $L^{\left(c\right)}\left(x\right)$, to be generally nonlinear, we studied their nontrivial fixed points. As mentioned earlier, Lorenz function L(x) has trivial fixed points (at x=0 and 1), while the Complementary Lorenz function $L^{\left(c\right)}\left(x\right)\equiv 1-L\left(x\right)$ has a nontrivial fixed point at x=k, the Kolkata index [49]. It also offers a tangible interpretation: *k*-index gives the fraction *k* of the total wealth possessed by the rich (1−k) fraction of the population and gives a quantitative generalization of the Pareto’s 80–20 law [55]. As discussed earlier, it can also be viewed as a normalized *h*-index of social inequality. Some unique features of Kolkata Index (k) may be noted: (a) Gini and other indices are mostly some average quantities based on the Lorenz function L(x), which has trivial fixed points. *k* is a fixed point of the Complementary Lorenz function $L^{\left(c\right)}\left(x\right)$ and, if one considers the simplest form of Lorenz function $L\left(x\right)=x^{2}$, then $k=(\sqrt{5}-1)/2$, inverse of the Golden Ratio [51]. (b) *k* gives the fraction of wealth possessed by the rich 1−k fraction of the population. As such, it provides a quantitative generalization of the Pareto’s 80–20 law (see, e.g., Reference [55]). The observed values of *k* index for most of the cases of social inequalities [50,51,52,53,54] seem to fall in the range 0.80-0.86 (though have much smaller values today for world economies, presumably because of various welfare measures). (c) *k*-index is equivalent to a normalized version of the Hirsch-Index (*h*). While *h* corresponds to the fixed point of the publication success rate (measured by the integer numbers of citations) falling off nonlinearly with number of papers by individual academicians, *k* corresponds to the fixed point (fraction) of 1−L(x), where L(x) gives the nonlinearly varying fraction of cumulative wealth possessed by the increasing (from poor to rich) fraction of population in any society.

### 3.3. Kinetic Exchange Model of Income and Wealth Distributions

We have discussed already in Section 2 some details of the Kinetic Exchange model of income and wealth distributions. In an ideal gas, in thermal equilibrium, the number density n(ϵ) of (Newtonian) particles (atoms or molecules) having kinetic energy ϵ is given by

(1) $$n\left(\epsilon \right)=g\left(\epsilon \right)f\left(\epsilon ,T\right)∼\sqrt{\left(\epsilon \right)}\exp\left(-\epsilon /\Delta \right),$$

where Δ is a constant, the density of states g(ϵ)∼$\sqrt{\epsilon }$ (coming from the counting of three-dimensional momenta vectors which correspond to the same kinetic energy) and f(ϵ)∼exp(−ϵ/Δ) (coming from stochastic, or entropy maximizing, scatterings between the particles, conserving their total kinetic energy). As discussed already in Section 2, to get the ideal gas equation of sate PV=NKT, where *P* and *V* denote, respectively, the pressure and volume of the gas at absolute temperature *T*, by calculating the pressure from the average transfer of momentum of the particles per unit area of the container (using Equation (1), Reference [7]), one identifies, Δ=KT.

Following similar arguments [7,8] (also see Reference [28] ), one gets (as discussed in Section 2) for the steady state distribution of the number n(m) of agents in the market with income or wealth *m*.

(2) $$n\left(m\right)=g\left(m\right)f\left(m\right)=c\exp\left(-m/\Delta^{\prime }\right),$$

where $f\left(m\right)∼\exp\left(-m/\Delta^{\prime }\right)$, with g(m) a constant *c* (unlike in expression (1)), and $\Delta^{\prime }$ as constants for the trading market. This is because, in a trading market, there is no production (growth) or decay, and the total amount of money (equivalent to energy in Kinetic theory of ideal gas), as well as the number of traders (buyers and sellers), remain fixed. Stochastic money exchanges in the trades involving indistinguishable buyers and sellers (who change their roles in different trades), keeping the buyer-seller total money in any trade to remain constant, lead to a distribution given by expression (2). This is also because there cannot be any equivalent of the particle momenta vectors for the agents in the market and hence the density of states g(m) here is a constant. One must also have $\int_{0}^{M}n\left(m\right)dm=N$, the total number of traders in the market, and $\int_{0}^{M}mn\left(m\right)dm=M$, the total amount of money. These give the effective temperature of the market $\Delta^{\prime }=M/N$, the average money in circulation in the market (economy).

As documented in several books and reviews (see, e.g., References [7,61,62]), the income or wealth distributions in any society have a Gamma function-like dip near zero income or wealth (unlike in the exponential distribution case discussed above, where the number density of pauper is the maximum). In addition, as is well known [62,63], the tail end of the distribution is known to be much more fat, described by the Pareto power law, and not by the thin exponentially decaying distribution.

As mentioned in the earlier section, following Saha and Srivastava’s indication in their book [7], we explored how the kinetic theory of trading markets indicated above could be extended to accommodate a Gamma-like distribution at the least and explore further to capture the Pareto tail of such a distribution, as well.

We noted that many of the economics text books, in their chapters on Trades, discuss the saving propensity of the traders (habit of saving a fraction of the income or wealth possessed by the trader and do trade with the rest). We immediately realized [9,10], if one introduces the saving propensity of each trader, so that each trader saves a fraction of their individual money (wealth) before the trade and allows (random) exchanges of the rest amount in the trade (keeping the total money or wealth, including the saved portions, conserved), the traders will never become paupers. Unlike in the random exchange case (as in kinetic theory of gases, where one trader may lose its entire amount of money or wealth to the other in any trade), here, to lose the entire amount of money acquired at any point of time, the trader has to lose every time after that as the trader continues the successive trades (and, consequently, the saved portion becomes infinitesimal). The number density of paupers (having zero wealth) will become zero for any non-vanishing saving fraction of the traders and the exponential distribution will become unstable and the resulting steady state distributions will capture the above mentioned desirable features. This is a non-perturbative result; any amount of saving by the traders will induce this feature.

With uniform saving, the exponential distribution collapses and the stable distribution becomes Gamma-like [9] (also see Reference [64] for a micro-economic derivation of the kinetic exchange equations from the Cobb-Dauglas utility maximization with money saving propensity of the traders, and Reference [65] for extended microeconomic formulation of Kinetic exchange models, having economic growths, by incorporating additional saving of the production in the utility maximization equation). The steady state distribution becomes initially Gamma-like but crossing over to Pareto-like power-law decay when traders have non-uniform saving propensities [10]. The saving propensity magnitudes determine the most-probable income (wealth) and the income (wealth) crossover point for Pareto tail of the distribution (see References [63,66,67] for details).

It may be mentioned in this connection that one kind of saving by the traders, considered early by our group (including the students Anirban Chakrabarti and Srutarshi Pradhan) can, in fact, lead to wealth condensation or extreme inequality. When two randomly selected traders agree to trade (in the so-called ’Yard Sale’ trade mode), such that the richer one among them will retain or save the extra money or wealth compared to that of her trade partner, the dynamics will eventually lead to aggregation of the entire amount of money or wealth in the hand of one trader, and the dynamics will stop. This happens because once any trader becomes pauper (loses entire amount of money or wealth), no other trader (with money) will engage in trade with her. Although this Yard Sale model has this uninteresting wealth condensation feature, it showed some interesting slow dynamics, and Anirban published that result [68]. Later, it was shown that inclusion of tax in the model, in the sense that a fraction of money is collected by the Government (non-playing member of the system) in every trade and, after some period of collections, redistributes the money among all (by investing on general social facilities, like road, hospital, etc., constructions, used equally by all in the society). Because of this general upliftment, the paupers come back to the trades and interesting steady state money distribution can emerge and such models of wealth distribution have become an active area of research (see, e.g., Reference [69] for a popular review on this development).

Kinetic model of gases and the kinetic theory is the first and extremely successful many-body theory in physics. Economic systems, markets in particular, are intrinsically many-body dynamical systems. Kinetic exchange models of markets may, therefore, be expected to provide the most successful models of market systems. In the kinetic exchange model, when one of the trader of a randomly chosen pair of traders is deliberately the poorest one at that instant of time (trade), the dynamics induces a self-organization in the market such that a ‘poverty line’ is spontaneously developed so that none of the trader remains below the emerged (self-organized) poverty threshold (see References [70,71] and references therein).

### 3.4. Statistics of the Kolkata Paise Restaurant Problems

Kolkata had, long back, very cheap fixed price ‘Paise Restaurants’ (also called ‘Paise Hotels’; Paise is, rather was, the smallest Indian coin). These ‘Paise Restaurant’ were very popular among the daily laborers in the city. During lunch hours, these laborers used to walk down (to save the transport costs) from their place of work to one of these restaurants. These restaurants would prepare every day a (small) number of such dishes, sold at a fixed price (Paise). If several groups of laborers would arrive any day to the same restaurant, only one group would get their lunch, and others would miss their lunch that day. There were no cheap communication means in those days (like mobile phones) for mutual communications, for deciding the respective restaurants. Walking down to the next restaurant would mean failing to report back to work on time! To complicate this collective learning and decision-making problem, there were indeed some well-known rankings of these restaurants, as some of them would offer tastier items compared to the others (at the same cost, Paise, of course), and people would prefer to choose the higher rank of the restaurant, if not crowded! This ‘mismatch’ of the choice and the consequent decision not only creates inconvenience for the prospective customers (going without lunch), would also mean ‘social wastage’ (excess unconsumed food, services, or supplies somewhere).

A similar problem arises when the public administration plans and provides hospitals (beds) in different localities, but the local patients prefer ‘better’ perceived hospitals elsewhere. These ‘outsider’ patients would then have to choose other suitable hospitals elsewhere. Unavailability of the hospital beds in the over-crowded hospitals may be considered as insufficient service provided by the administration, and, consequently the unattended potential services will be considered as social wastage.

This kind of games [72] (see References [20,73] for recent reviews), anticipating the possible strategies of the other players and acting accordingly, is very common in society. Here, the number of choices need not be very limited (as in standard binary-choice formulations of most of the games, for example, in Minority Games [19,20,73]), and the number of players can be truly large! In addition, these are not necessarily one shot games, rather the players can learn from past mistakes and improve on their selection strategies for choosing the next move. These features make the games extremely intriguing and also versatile, with major collective or emerging social structures, not comparable to the standard finite choice, non-iterative games among finite number of players. Such repetitive collective social learning for a community sharing past knowledge for the individual intention to be in minority choice side in successive attempts are modeled by the ‘Kolkata Paise Restaurant’ (KPR) problem or, in short, by the ‘Kolkata Restaurant’ problem.

KPR is a repeated game, played among a large number of players or agents having no simultaneous communication or interaction among themselves. In KPR, the prospective players (customers/agents) choose from restaurants each day (time) in parallel decision mode, based on the past (crowd) information and their own (evolved or learned) strategies. There is no budget constraint to restrict the choice (and hence the solutions). Each restaurant has the same price for a meal but having a different rank, agreed upon by all the customers or players.

For simplicity, we may assume that each restaurant can serve only one customer (generalization to any fixed number of daily services for each would not change the complexion of the problem or game). If more than one customer arrives at any restaurant on any day, one of them is randomly chosen and is served, and the rest do not get meal that day. Information regarding the prospective customer or crowd distributions for the earlier days (up to a finite memory size) is made available to everyone. Each day, based on own learning and the developed (often mixed) strategies, each customer chooses a restaurant independent of the others. Each customer wants to go to the restaurant with the highest possible rank while avoiding a crowd so as to be able to get the meal there. Both from individual success and social efficiency perspective, the goal is to ‘learn collectively’ to utilize effectively the available resources.

The KPR problem seems to have a trivial solution: suppose that somebody, say a dictator (who is not a player), assigns a restaurant to each person the first day and asks them to shift to the next restaurant cyclically, on successive days. The fairest and most efficient solution: each customer gets food on each day (if the number of plates or choices is the same as that of the customers or players) with the same share of the rankings as others, and that too from the first day (minimum evolution time). This, however, is NOT a true solution of the KPR problem, where each customer or agent decides on his or her own every day, based on complete information about past events. In KPR, the customers try to evolve learning strategies to eventually to arrive at the best possible solution (close to the dictated solution indicated above). The time for this evolution needs also to be optimized; for example, a very efficient strategy, having convergence time which grows with the number of players (even linearly), is unsuitable for most of the social games, as our life-span is finite, and (in a democracy) the number of players or competitors cannot be restricted or bounded.

There have been many limiting formulations and studies using tricks from statistical physics and quantum physics (see, e.g., References [20,73,74,75,76,77,78,79,80,81]) and generalizations in computer science (see, e.g., Reference [82]) and mobility (vehicle on hire) markets (see, e.g., References [83,84] ). We will present briefly in the next section some specific results of a new study on the nature phase transition and resource utilization in KPR with number of customers less (still very large) than the number of restaurants.

## 4. Some New Results for Statistics of the KPR Problem

Here, we consider the case where λN agents decide to choose among *N* available resources (for λ < 1). Every day each restaurant prepares one dish for lunch and serve it to the visitor. If, on some day, any restaurant is visited by more than one agent, then one of them is randomly chosen and served the prepared dish; the rest leave and have to starve for that day. Thus, every agent is required to make her choice such that the chosen restaurant will be visited alone by her (at most one agent arriving each restaurant) to assure her lunch that day. As λ is less than unity here, a fraction (1−λ) of restaurants will any way go vacant any day. Additionally, a fraction (1−f(t)) of restaurants will go vacant on day (t) because of overcrowding at some restaurants due to fluctuations in choices of the prospective customers. On any day *t*, the average social success factor *f* for the agents, can be measured as

(3) $$f\left(t\right)=\sum_{i=1}^{N}\left[\delta \left(n_{i}\left(t\right)\right)/\lambda N\right],$$

with δ(n)=1 for n=1 and δ(n)=0 otherwise; $n_{i}\left(t\right)$ denotes the number of agents arriving at the *i*th (rank) restaurant on day *t*. [1−f(t)] gives the fraction of wastage due to fluctuation of choices and [(1−λ)+(1−f(t)] gives the fraction of restaurants not visited by any agent on day *t*. The goal is to achieve f(t) = 1 preferably in finite convergence time (τ), i.e., for *t*≥τ, or at least as t→∞.

As usual, a dictated solution is extremely simple and efficient: A dictator asks everyone to form a queue for visiting the restaurants in order of their respective positions in the queue and then asks them to shift their positions by one step (rank) in the next day (assuming periodic boundary condition). Everyone gets the food and the steady state (*t*-independent) social utilization fraction f=1. This is true even when the restaurants have ranks (agreed by all the agents or customers).

However, in democratic set-up, this dictated solution is not acceptable and the agents or players are expected to evolve their strategy to make the best minority choice independently (without presence of any dictator), using the publicly available information about the past record of crowd sizes in different restaurants, such that each arrives alone there in the respective restaurant and gets the dish. The more successful such collective learning, the more is the aggregated utilization fraction *f*. Earlier studies (see e.g., References [20,72,74,85,86,87,88] strategies for KPR game. Recently authors in Reference [81] have proposed two such stochastic strategies (strategy I and strategy II) where the agents collectively learn to make their decisions utilizing the publicly available history of crowd size of the last day’s chosen restaurant. Below, we briefly discuss them.

**Strategy I:**

On day *t*, an agent goes back to her last day’s visited restaurant *k* with probability

(4) $$p_{k}^{\left(I\right)}\left(t\right)={\left[n_{k}\left(t-1\right)\right]}^{-\alpha },\;\alpha >0.$$

If $n_{k}\left(t-1\right)>1$, each of the $n_{k}\left(t-1\right)$ agents or players try to arrive at the same *k*-th restaurant next day *t* with the above probability and chooses a different one $\left(k^{{}^{\prime }}\neq k\right)$ among any of the neighboring restaurants $n_{r}$ on day *t*, with probability

(5) $$p_{k^{{}^{\prime }}}^{\left(I\right)}\left(t\right)=\left(1-p_{k}^{\left(I\right)}\left(t\right)\right)/n_{r}.$$

**Strategy II:**

On day *t*, an agent tries to go back to the same restaurant as chosen the earlier day (t−1) with probability

(6) $$p_{k}^{\left(II\right)}\left(t\right)=1,\;\mathrm{if}\;n_{k}\left(t-1\right)=1\;\;\mathrm{and}$$

(7) $$p_{k^{{}^{\prime }}}^{\left(II\right)}\left(t\right)=p<1,\;\mathrm{if}\;n_{k}\left(t-1\right)>1$$

for choosing any of the $n_{r}$ neighboring restaurants ($k^{{}^{\prime }}\neq k$).

### 4.1. Numerical Results

We have numerically studied the steady state dynamics of the KPR game where every day λN agents decide which restaurant to choose and visit among *N* restaurants following both the strategies I and II. We consider here infinite dimensional arrangement for restaurants, where the number of nearest neighboring restaurants $n_{r}$ to each is (N−1), and the cost to visit any of them is the same for all the time. The maximum social utilization *f* obtained from Equation (3) (from the point of view of agents or players) will be denoted further by $f^{a}$. Each day (iteration), parallel choice decisions by each are processed (following either strategy I or II) and used to compute $f^{a}$. Steady state is identified as the state when $f^{a}$ does not change (within a predefined error margin) for the next (say, hundred) iteration.

**Figure 6:**
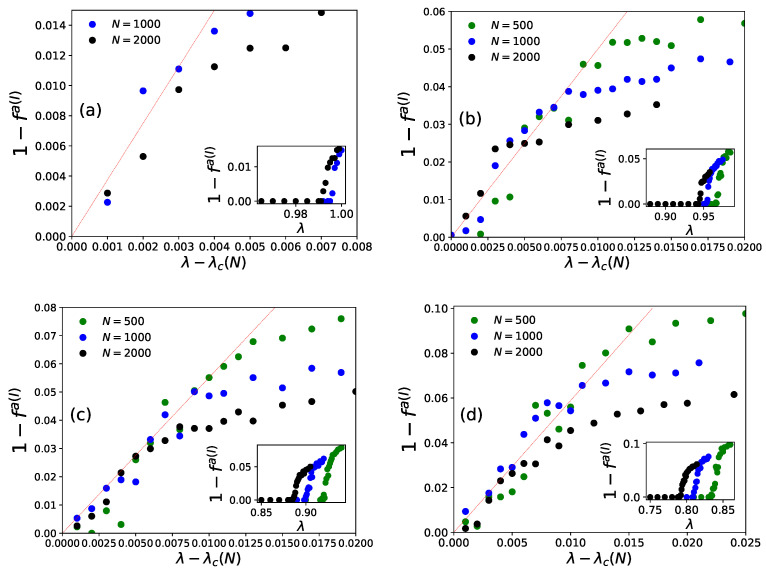
Plots of $\left(1-f^{a(I)}\right)$ against $\lambda -\lambda_{c}\left(N\right)$ following strategy I at (**a**) α=0.05, (**b**) α=0.25, (**c**) α=0.5, (**d**) α=1.0. A power law holds for $\left(1-f^{a(I)}\right)∼{\left(\lambda -\lambda_{c}\left(N\right)\right)}^{\beta }$, where β=1.0±0.05. The insets show direct relationship between $\left(1-f^{a(I)}\right)$ and λ (for strategy I).

**Figure 7:**
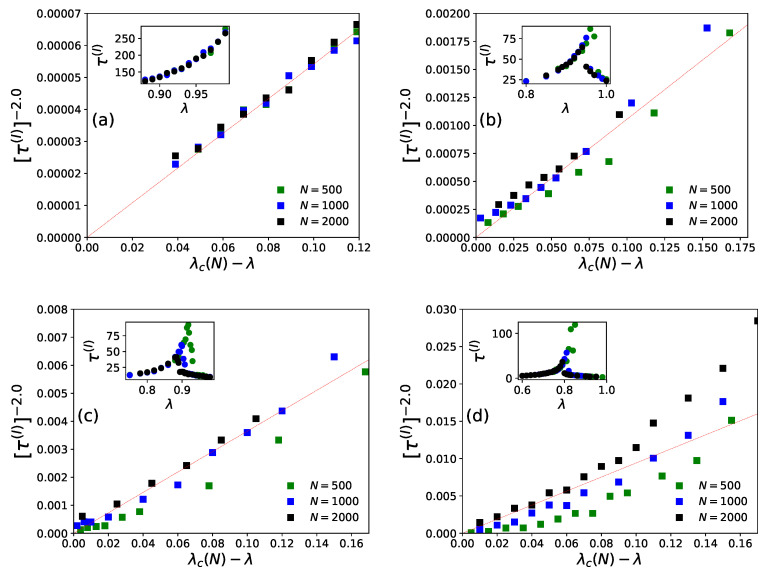
Plots of steady state convergence time $\tau^{\left(I\right)}$ from strategy I against $\lambda_{c}\left(N\right)-\lambda$ at (**a**) α=0.05, (**b**) α=0.25, (**c**) α=0.5, (**d**) α=1.0. A power law holds for $\tau^{\left(I\right)}∼{\left(\lambda_{c}\left(N\right)-\lambda \right)}^{-\gamma }$, where γ=0.5±0.05. The insets plot direct relationship between $\tau^{\left(I\right)}$ and λ for different system sizes (for strategy I), also showing the variation of λ as α increases.

**Figure 8:**
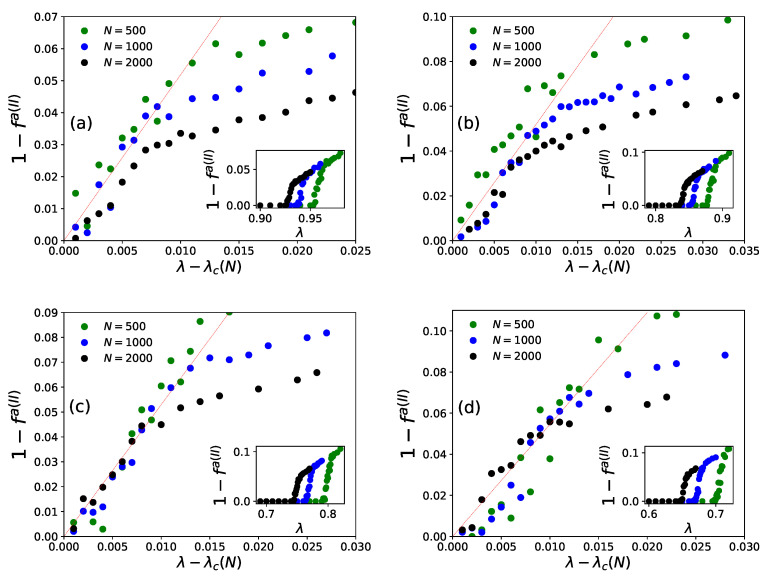
Plots of $\left(1-f^{a(II)}\right)$ versus $\lambda -\lambda_{c}\left(N\right)$ following strategy II at (**a**) p=0.8, (**b**) p=0.6, (**c**) p=0.4, (**d**) p=0.2. A power law holds for $\left(1-f^{a(II)}\right)∼{\left(\lambda -\lambda_{c}\left(N\right)\right)}^{\beta }$ with β=1.0±0.05. The insets show direct relationship between variations of $\left(1-f^{a(II)}\right)$ against λ (for strategy II).

**Figure 9:**
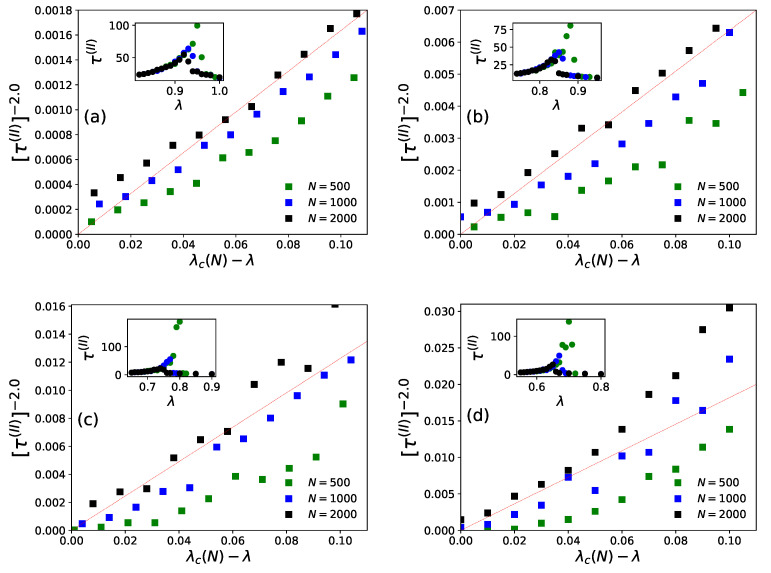
Plots of steady state convergence time $\tau^{\left(II\right)}$ against $\lambda_{c}\left(N\right)-\lambda$ following strategy II at (**a**) p=0.8, (**b**) p=0.6, (**c**) p=0.4, (**d**) p=0.2. A power law holds for $\tau^{\left(II\right)}∼{\left(\lambda_{c}\left(N\right)-\lambda \right)}^{-\gamma }$ with γ=0.5±0.07. The insets give direct relationship between $\tau^{\left(II\right)}$ and λ for different system sizes (for strategy II), also showing the variation of λ as *p* decreases.

**Figure 10:**
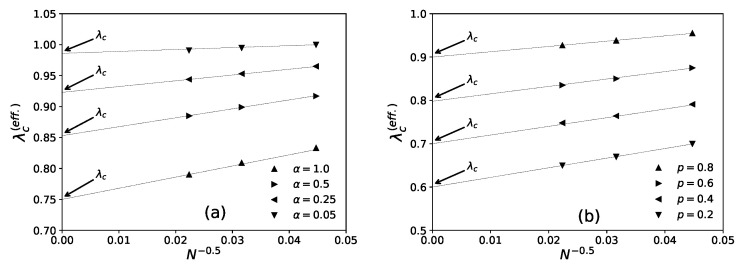
Extrapolation study of the effective finite size critical density of agents $\lambda_{c}\left(N\right)$. The system size dependence is numerically fitted to $\frac{1}{\sqrt{N}}$ and we estimate $\lambda_{c}$ from $\lambda_{c}\equiv \lambda_{c}\left(N\to \infty \right)$. The extrapolated values of $\lambda_{c}$ are 0.99,0.92,0.85,0.75 for α=0.05,0.25,0.5,1.0 (strategy I) (**a**), and are 0.9,0.8,0.7,0.6 for p=0.8,0.6,0.4,0.2 (strategy II) (**b**).

On day *t*, $n_{i}\left(t-1\right)$, agents decide to revisit last day’s visited restaurant (i) with probability $p_{k}^{\left(I\right)}\left(t\right)$ (Equation (4)) or probability $p_{k}^{\left(II\right)}\left(t\right)$ (Equation (6)), or else choose any other $\left(k^{\prime }\neq k\right)$ from among any of the (N−1) neighboring restaurants for both the strategies (Equations (5) and (7)). After the system stabilizes, ($f^{a}\left(t\right)$ becomes practically independent of *t*, the average statistics of $f^{a}\left(t\right)$ are noted as $\left[f^{a(I)}\right]$ or $\left[f^{a(II)}\right]$, respectively, for strategies I and II. We find the power law fits for the steady state wastage fraction $\left(1-f^{a(I)}\right)∼\left(1-f^{a(II)}\right)∼{\left(\lambda -\lambda_{c}\left(N\right)\right)}^{\beta }$ with β=1.0±0.05 (see Figure 6 and Figure 7) and $\tau^{\left(I\right)}∼\tau^{\left(II\right)}∼{\left(\lambda_{c}\left(N\right)-\lambda \right)}^{-\gamma }$ with γ=0.5±0.07 (see Figure 8 and Figure 9) in both of the strategies I and II. Varying λ, the steady state results of $f^{a}$, τ for different system sizes (N=500,1000,2000), with α=0.05,0.25,0.5,1.0 in strategy I or p=0.2,0.4,0.6,0.8 in strategy II are considered here. All simulations are done taking maximum N=2000 with numbers of iteration/run of order $10^{6}$. For finite system sizes, the effective critical points $\lambda_{c}\left(N\right)$ (where $f^{a}$ becomes unity or τ reaches its peak value) obtained numerically for different system sizes (N) and are analyzed using finite size scaling method in Figure 10.

It may be mentioned that, in general, for the estimation of errors in the exponents β and γ in Figure 6, Figure 7, Figure 8 and Figure 9, we tried linear fits (without any intercept) for logy versus logx, using best fits mostly for the intermediate range data points for all *N* values until they start deviating (due to extreme fluctuations near $\lambda =\lambda_{c}$ and towards their saturation values for λ approaching unity [74,81]) and anticipating their universal mean field values in this infinite dimensional system. From the slopes of these best fit lines for different α or *p* values, we extract the universal exponent values and their standard deviations. We give this higher error in the estimate of the unified (and universal) estimate of γ.

### 4.2. Summary

KPR is a multi-agent multi-choice repeated game where players try to learn from their past successes or failures, utilizing publicly available information on the crowd sizes at different restaurant in the past to decide which restaurant to visit that day such that she would be alone there for being served the only prepared dish. Here, asymmetric case such that λN(λ<1) agents are considered against *N* restaurants, for sufficiently large *N*. End of each day (iteration), we have measured social utilization for agents $f^{a}\left(t\right)=\sum_{i=1}^{N}\left[\delta \left(n_{i}\left(t\right)\right)/\lambda N\right]$ where $n_{i}\left(t\right)$ denotes number of customers visiting *i*th restaurant on day *t*.

As shown in Figure 6 (for strategy I) and Figure 8 (for strategy II), the social wastage fraction $\left(1-f^{a}\right)$ vanishes at the effective critical point $\lambda_{c}\left(N\right)$ with the critical exponent β value near unity. In addition, from Figure 7 (for strategy I) and Figure 9 (for strategy II), we see that the the convergence or relaxation time *t*, required for $f^{a}$ to stabilize, divergence near the same critical points $\lambda_{c}\left(N\right)$ for the respective strategies, with the exponent γ value about 1/2. Additionally, the finite size scaling analysis $\lambda_{c}\left(N\right)∼\lambda_{c}+const.N^{-1/(d\nu )}$, where $\lambda_{c}$ corresponds to $\lambda_{c}\left(N\right)$ for *N* going to infinity and *d* corresponds to the effective dimension, suggests the effective correlation length exponent dν value to be around 2 for both the strategies, as expected for such mean field (infinite range systems).

In Reference [81], we have studied the dynamics of the KPR game following the same two strategies for the case λ=1. For λ=1.0, where the critical points $\lambda_{c}$ (for both the strategies) vanish, the universality class (values of the critical exponents β and γ were observed to be distinctly different, and this point needs further investigations. We may, however, note that, since at λ=1, the number of both agents and restaurants are same (*N*), full social utilization (where $f^{a}=1=f^{r}$, occurring at $\alpha =0_{+}$ for strategy I and at $p=1_{-}$ for strategy II) induces an additional frustrating constraint in the collective choice dynamics involved here.

The KPR game models have been extended already and used to study real life problems, like resource allocation in Internet of Things [82], vehicle for hire [83], matching in mobility markets [84], etc. We hope the KPR game models will be utilized much more effectively in the context of much wider practical areas of collective learning dynamics and choices.

## 5. Future of Econophysics: Some Perspective

One often says that the main purpose of economic activity is to optimize the limited funds of labor and capital, natural and technical resources and capital resources, to satisfy our (practically) unlimited needs. “Economic science is therefore the science of efficiency, and as such, it is a quantitative science.” [89] (also see Reference [90]). We have already argued [14] in Section 2 that epistemologically economics belongs to natural science (and not mathematics). It begins with observation which are to be analyzed using logic or mathematics and eventually should end in observation, as in all natural sciences. Since 1990s, most Universities of the world offer Science Graduation degrees (Bachelor of Science or Master of Science degrees) in economics (in addition to Bachelor of Arts or Master of Arts from Fine Arts or Humanities Departments).

Robert Solow [91] pointed out that, in the 1940s, economics had been basically a descriptive and institutional subject for a ‘gentleman scholar’. The textbooks of those days were ‘civilized’ and discursive. … “Formal analysis were minimal and it made economics the domain of intuitive economists”. He concluded his summary of the state of economics near the end of the 20th century “with a paraphrase of Oscar Wilde’s description of a fox hunt-‘the unspeakable in pursuit of the inedible’-saying that perhaps economics was an example of ‘the over-educated in pursuit of the unknowable’.” [91]. Despite the ongoing controversies today in the field of economics, the “New Millennium economists are far more comfortable with what they do after the changes in the structure and content of economics over the last half century” [92]. The root cause of these changes have been identified by Colander [92] to be due to the rise of Complexity Science since early 1980s. In fact, concepts from physics had continually been absorbed into the main stream economic formulation of ideas and models. As Venkat Venkatasubramanian noted in his recent book [93], “Concepts such as equilibrium, forces of supply and demand, and elasticity reveal influence of classical mechanics on economics. The analytical model of utility-based preferences can be traced back to Daniel Bernoulli, the great Swiss mathematical physicist from nineteenth century. One of the founders of neoclassical economics, Irving Fisher, was trained under the legendary Yale physicist, Jisiah Willard Gibbs, a co-founder of the discipline of statistical mechanics. Similarly, Jan Tinbergen, who shared the first Nobel Prize in Economics in 1969, was the doctoral student of the great physicist Paul Ehrenfest at Leiden University”.

Indeed, more specifically as discussed in Section 2, we would like to correlate these changes to occur following the successful development in econophysics of the Simulated Annealing technique [22] in 1983 for Traveling Salesman type multi-variable optimization problems, and other successive developments in econophysics of analyzing correlations in stock prices (see, e.g., References [3,21]) or the kinetic exchange modelings of income and wealth distributions (see, e.g., References [28,63]). The statistical physics of TSP, as an example of successful developments in econophysics, had already been introduced in our 2010 econophysics textbook [32], which has been the only ‘suggested textbook’ (since inception in 2012) of the formal course on econophysics, offered (by Diego Garlaschelli) at the Physics Department of the Leiden University (see the course prospectus for 2012–2013 through that of 2020–2021 [94]), where one of the first Nobel-laureates in economics Jan Tinbergen came from.

Econophysics has come as an exceptional development in interdisciplinary sciences (see, e.g., Reference [95] for a popular exposition on this development). Historically, economics, more specifically social sciences, belonged to the Humanities departments and not of Science. For earlier interdisciplinary developments of Astrophysics, Biophysics, or Geophysics, the scenario and ambiance had been quite different. The mother departments had been parts of the same science schools and even the corresponding resources, like books, journals, and also the faculty, had strong overlaps and could be shared. The marriage negotiations for Econophysics have been difficult, though extremely desirable and natural; as the saying goes: “marriage between the King of natural sciences with the Queen of social sciences!”

Regular interactions and collaborations between the communities of natural scientists and social scientists are, however, rare, even today! Though, as mentioned already, interdisciplinary research papers on econophysics and sociophysics are regularly being published at a steady and healthy rate, and a number of universities (including Universities of Bern, Leiden, London, Paris, and Tufts University) are offering the interdisciplinary courses on econophysics and sociophysics, not many clearly designated professor positions, or other faculty positions for that matter, are available yet (except for econophysics in Universities of Leiden and London). Neither are there designated institutions on these interdisciplinary fields, nor separate departments or centers of studies for instance. Of course, there have been several positive and inspiring attempts and approaches from both economics and finance side (see, e.g., References [96,97], along with a number of those [66,67,98,99,100] from physics, which have already been appreciated in the literature). Indeed, the thesis [101] in August 2018, Department of History and Philosophy of Science, University of Cambridge, by financial economist Christophe Schinckus (one of the co-editors of this special issue), says that “In order to reconstruct the subfield of econophysics, I started with the group of the most influential authors in econophysics and tracked their papers in the literature using the Web of Science database of Thomson-Reuters (The sample is composed of: Eugene Stanley, Rosario Mantegna, Joseph McCauley, Jean-Pierre Bouchaud, Mauro Gallegati, Benoît Mandelbrot, Didier Sornette, Thomas Lux, Bikas Chakrabarti and Doyne Farmer). These key authors are often presented as the fathers of econophysics simply because they contributed significantly to its early definition and development. Because of their influential and seminal works, these scholars are actually the most quoted authors in econophysics. Having the 10 highest quoted fathers of econophysics as a sample sounds an acceptable approach to define bibliometrically the core of econophysics”. In addition, the entry on ‘Social Ontology’ in The Stanford Encyclopedia of Philosophy [15], as discussed in Section 2, confirms positive impact of such econophysics and sociophysics research studies on the overall modern philosophical outlook of social sciences.

**Figure 11:**
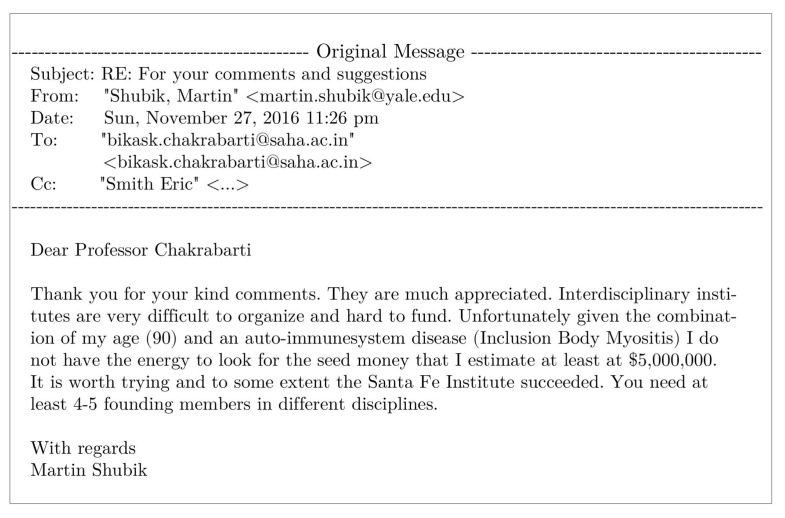
The first part of the email conversation between (late) Martin Shubik and BKC. Second part (email from BKC; appended to this part) is continued in Figure 12. The precise suggestions made in this immediate response indicate Shubik’s prior plan for such ‘interdisciplinary institutes’ in economics.

**Figure 12:**
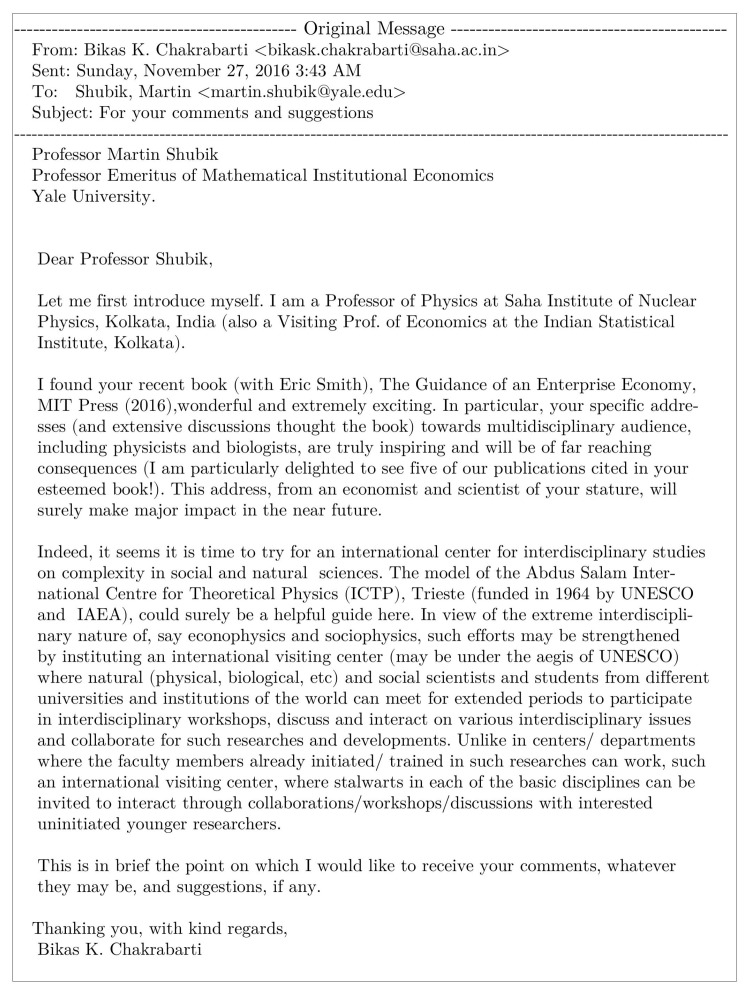
Email conversation in the end of 2016 between (late) Martin Shubik and BKC regarding interdisciplinary developments in economics and the possibility of setting up an International Center for Interdisciplinary Studies on Complexity in Social Sciences. This email from BKC was appended to the response email (Figure 11) from Shubik. The (Yale) date and time mark in the mail-header (and that for BKC’s in Figure 11, on arrival in Kolkata) indicate hardly any time gap between the two and the readiness with the precise suggestions indicate Shubik’s prior thinking in similar line.

We may note, however, a recently published highly acclaimed massive (580 page) book [96] on economics (‘landmark volume’, said E. Roy Weintraub, ‘creative, elegant and brilliant work’, said W. Brian Arthur and ‘written by master economists’, said D. Colander) by (Late) Martin Shubik (Ex-Seymour Knox Professor Emeritus of Mathematical Institutional Economics, Yale University and Santa Fe Institute) and Eric Smith (Santa Fe Institute) discussed extensively on econophysics approaches and in general on the potential of interdisciplinary research studies inspired by the developments in natural sciences. Getting somewhat excited, I wrote to Martin Shubik in late 2016 that their book can also serve as an outstanding ‘white-paper’ document in favor of a possible Proposal for an International Center for Interdisciplinary Studies on Complexity in Social Sciences. He immediately responded and gave his impression about the difficulties involved and indicated very briefly about the minimal financial and structural requirements (both my letter to him and his response is appended below (Figure 11 and Figure 12).

This ready and specific comments by Shubik clearly suggests that he actually had thought about the need of such an International Center for fostering interdisciplinary research which needs to be more inclusive than, for example, the Santa Fe Institute. The model of the Abdus Salam International Center for Theoretical Physics (ICTP), Trieste (funded by UNESCO and IAEA), was considered to provide helpful guidance for us here. It was contemplated, if an ICTP-type interdisciplinary research institute could be initiated for research studies on econophysics and sociophysics (see, e.g, Reference [102]). Though Shubik (who died in 2018 at the age of 92) agreed also to be one of its founding members, we could not make any progress yet. We may also note that Dirk Helbing and colleagues have been trying for an European Union funded ‘Complex Techno-Socio- Economic Analysis Center’ or ‘Economic and Social Observatory’ for the last decade or so (see Ref. [103] containing the White Papers arguing for their proposed project). We are also aware that Indian Statistical Institute had taken a decision to initiate a similar Center in India (see ‘Concluding Remarks’ in Reference [104]).

Hope, some such international visiting centers will come up soon and with them the spread of such interdisciplinary ideas will achieve more coherence and will lead to major success in such research studies.

## Data Availability

Not applicable.
